# Response Audit of an Internet Survey of Health Care Providers and Administrators: Implications for Determination of Response Rates

**DOI:** 10.2196/jmir.1090

**Published:** 2008-10-16

**Authors:** Mark J Dobrow, Margo C Orchard, Brian Golden, Eric Holowaty, Lawrence Paszat, Adalsteinn D Brown, Terrence Sullivan

**Affiliations:** ^9^Cancer Care OntarioTorontoONCanada; ^8^Health System StrategyOntario Ministry of Health and Long-Term CareTorontoONCanada; ^7^Institute for Clinical Evaluative SciencesTorontoONCanada; ^6^Public Health SciencesUniversity of TorontoTorontoONCanada; ^5^Population Studies and SurveillanceCancer Care OntarioTorontoONCanada; ^4^Collaborative for Health Sector StrategyTorontoONCanada; ^3^Rotman School of ManagementUniversity of TorontoTorontoONCanada; ^2^Health PolicyManagement and EvaluationUniversity of TorontoTorontoONCanada; ^1^Cancer Services & Policy Research UnitCancer Care OntarioTorontoONCanada

**Keywords:** Health care surveys, Internet, survey methodology

## Abstract

**Background:**

Internet survey modalities often compare unfavorably with traditional survey modalities, particularly with respect to response rates. Response to Internet surveys can be affected by the distribution options and response/collection features employed as well as the existence of automated (out-of-office) replies, automated forwarding, server rejection, and organizational or personal spam filters. However, Internet surveys also provide unparalleled opportunities to track study subjects and examine many of the factors influencing the determination of response rates. Tracking data available for Internet surveys provide detailed information and immediate feedback on a significant component of response that other survey modalities cannot match. This paper presents a response audit of a large Internet survey of more than 5000 cancer care providers and administrators in Ontario, Canada.

**Objective:**

Building upon the CHEcklist for Reporting Results of Internet E-Surveys (CHERRIES), the main objectives of the paper are to (a) assess the impact of a range of factors on the determination of response rates for Internet surveys and (b) recommend steps for improving published descriptions of Internet survey methods.

**Methods:**

We audited the survey response data, analyzing the factors that affected the numerator and denominator in the ultimate determination of response. We also conducted a sensitivity analysis to account for the inherent uncertainty associated with the impact of some of the factors on the response rates.

**Results:**

The survey was initially sent out to 5636 health care providers and administrators. The determination of the numerator was influenced by duplicate/unattached responses and response completeness. The numerator varied from a maximum of 2031 crude (unadjusted) responses to 1849 unique views, 1769 participants, and 1616 complete responses. The determination of the denominator was influenced by forwarding of the invitation email to unknown individuals, server rejections, automated replies, spam filters, and ‘opt out’ options. Based on these factors, the denominator varied from a minimum of 5106 to a maximum of 5922. Considering the different assumptions for the numerator and the denominator, the sensitivity analysis resulted in a 12.5% variation in the response rate (from minimum of 27.3% to maximum of 39.8%) with a best estimate of 32.8%.

**Conclusions:**

Depending on how the numerator and denominator are chosen, the resulting response rates can vary widely. The CHERRIES statement was an important advance in identifying key characteristics of Internet surveys that can influence response rates. This response audit suggests the need to further clarify some of these factors when reporting on Internet surveys for health care providers and administrators, particularly when using commercially available Internet survey packages for specified, rather than convenience, samples.

## Introduction

There has been perceptible growth in the popularity of Internet surveys over the last decade. However, Internet survey modalities often compare unfavorably with traditional survey modalities, particularly with respect to response rates [1-7]. Response to Internet surveys can be affected by the distribution options and response/collection features employed as well as the existence of automated (out-of-office) replies, automated forwarding, server rejection, and organizational or personal spam filters. However, Internet surveys also provide unparalleled opportunities to track study subjects and examine many of the factors influencing the determination of response rates. Tracking data available for Internet surveys provides detailed information and immediate feedback on a significant component of response that other survey modalities cannot match [9]. This information generates questions about the appropriateness of traditional methods for determining response rates that may affect the comparability of results between Internet and mail/telephone surveys. This paper presents a response audit of a large Internet survey of more than 5000 cancer care providers and administrators in Ontario, Canada. Building upon the CHEcklist for Reporting Results of Internet E-Surveys (CHERRIES) [10], which is similar to other checklists for reporting on research such as CONSORT (for randomized trials) or QUORUM (for systematic reviews), the main objectives of the paper are to (a) assess the impact of a range of factors that influence response rates for Internet surveys and (b) recommend steps for improving published descriptions of Internet survey methods.

## Methods

As part of a study to measure the coordination and integration of cancer services, we developed the Cancer Services Integration (CSI) Survey [11]. The intent was to administer the survey to over 5000 physicians, other clinicians and a range of managers and administrators based at comprehensive cancer centers, teaching hospitals, community hospitals, and community care access centers across Ontario involved in the organization and/or delivery of cancer services.

After considering the relative impact on cost, response rates, and survey design [1,3,5-8,12-15], the decision was made to administer the CSI Survey via the Internet. A plethora of vendors provide ‘canned’ Web-based survey tools for administering Internet surveys [16]. While some features do vary, as do the fixed and variable rates charged for the service, the basic design options available are similar across Internet survey vendors. Therefore, based primarily on cost considerations, SurveyMonkey.com was selected as the vendor through which we would conduct the Internet survey.

### Internet Survey Options

The key Internet survey options that influence the determination of response rates can be categorized into 2 main groups: (a) distribution/list management options and (b) response/collection options.

### Distribution/List Management Options

Internet surveys can be distributed in many different ways, with the most typical involving email invitations to a specified sample or Web-based pop-up invitations to a convenience sample [8,17,18]. For this survey, we planned to distribute the survey to a specified sample of study subjects via an email invitation that included a link to a Web-based survey. Internet survey vendors, such as SurveyMonkey.com, usually provide two main options for distributing Internet surveys via email. The first and most basic option involves the creation of a generic survey Web link that can be copied and inserted into any email message. The email message with the generic survey Web link can then be sent to study subjects for which email addresses are available. When invited study subjects click on the link, they are taken to the Web-based survey where they can complete the survey based on the response/collection settings discussed below. While this option ensures confidentiality for participants, from a research perspective, the key limitation is that no information on individual response status is collected (eg, you cannot determine whether a specific study subject initiated a response, completed the survey or declined to participate). Furthermore, the email with the generic survey Web link can be forwarded to an infinite number of other email addresses, allowing other individuals to complete the survey with researchers unable to determine which respondents were part of the original sample and which were not (eg, it is possible to have more responses than intended study subjects).

The second option addresses most of these limitations by using a list management feature. For SurveyMonkey.com, this involves importing a list of email addresses into a secure, online database and then using the list management feature to automatically distribute a customizable email that contains an individual-specific survey Web link to all study subjects in the list. This list management option provides constantly updated information on the response status (eg, responded, no response, declined to participate, etc.) of each individual in the list. This option allows automated reminder messages to be generated and directed to specific subsets of the sample (eg, only to non-responders). There are a couple of limitations to the list management feature, however. We found the response status was usually, but not always, accurate (eg, in some cases, the response status indicated that specific individuals had not responded when in fact they had done so). Also, information on incomplete responses is not particularly useful for sending out reminder messages as no distinction is made between a respondent who completes a very small portion of the survey (eg, one question) and another respondent who completes a large portion of the survey (eg, all but the last question). Despite these limitations, we selected the list management feature, which from a research perspective, is preferable for distributing an Internet survey to a specified sample.

### Response/Collection Options

In addition to distribution/list management options, there are several response/collection options that enable or limit a respondent’s access to an Internet survey. These options can (a) affect the number of responses that can be entered by an individual respondent (eg, when a respondent clicks on the survey Web link, they can be taken to a blank survey, or, if they have already initiated their response, can be taken to the last question they responded to), (b) affect the number of sittings upon which the survey can be completed (eg, require that the survey must be completed in one sitting vs multiple sittings), (c) affect the ability to move backwards and forwards though the survey to edit/change responses, and (d) affect the ability to have multiple respondents respond from the same computer. For example, at the time of our survey launch (February 2007) SurveyMonkey.com had four main response/collection options when using the list management option, described on the vendor’s website as follows:

1. *One Response per Respondent* – After completing the survey, respondents will be prevented from entering additional responses. Respondents that return to a survey later will be able to edit their existing answers. Respondents that return to an incomplete survey will be taken to the point that they left off.
2. *One Response per Respondent (Forward Only)* – After completing the survey, respondents will be prevented from entering additional responses. In addition, respondents are prevented from backing up to edit their existing answers. Respondents that return to an incomplete survey will be taken to the point that they left off.
3. *Multiple Responses per Respondent* – After completing the survey, respondents will be allowed to enter an additional response. Respondents that return to an incomplete survey will be taken to the point that they left off.
4. *Multiple Responses per Respondent (Shared Computer)* – After completing or exiting the survey, respondents will be allowed to enter additional responses. Once respondents leave the survey, their answers are considered finished and cannot be edited. Useful for computer labs and tradeshow kiosks.

These response/collection options are critical as they affect the prevalence of duplicate and/or incomplete responses in the final data set. As a significant portion of our sample, particularly clinic nurses and radiation therapists, often share access to computers with Internet access at work, the response/collection option needed to accommodate multiple respondents per computer. We conducted extensive pre-testing of the four SurveyMonkey.com options combined with the list management feature to determine the impact on respondents’ access to partially complete responses, response confidentiality, and data capture. This testing revealed several issues. Of particular concern, options (1), (2), and (3) did not consistently protect response confidentiality. These options, which each allowed a respondent to return to the last question completed, also allowed other individuals, if they had been forwarded the original email invitation, to see the original respondent’s responses in some situations. Options (1)) and (2) did not allow duplicate responses; however our testing revealed situations where original, but not fully complete, responses could be overwritten by subsequent responses, whether by the same individual or another individual forwarded the email invitation. Ultimately, option (4) was selected as it was the only option that consistently protected response confidentiality in shared computer contexts and did not allow initial responses to be overwritten.

The main concern with option (4) was that it allowed multiple responses per unique email address. While the invitation email with survey Web link could be forwarded to other email addresses, based on option (4), all individuals who accessed the survey through that individual-specific Web link would have their responses linked to the original individual-specific email address. This creates uncertainty regarding whether a duplicate response originated from the intended study subject or from other unintended individuals. Also, option (4) did not allow respondents to access prior incomplete responses (rather a blank survey was accessed every time the Web link was selected), therefore we instructed study subjects to make every attempt to complete the entire survey in one sitting (approximately 10-15 minutes).

### Survey Distribution and Response Audit

Using the list management feature and response/collection option (4) allowing multiple responses per respondent from a shared computer, we imported 5636 email addresses into the SurveyMonkey.com list management database and created custom email invitations for each regional cancer program in Ontario. The initial automated invitation email was sent to all individuals in the list management database on 26 February 2007 with 3 automated reminder emails sent out to all individuals in the list management database with a response status of ‘no response’. To reduce the impact of forwarding of the invitation email to unidentified individuals, the first question on the survey asked the respondent how they accessed the survey. Those individuals who indicated that they did not receive the invitation email from csi.survey@cancercare.on.ca were asked to contact the study’s research coordinator who would send out an original invitation email if the individual fit the sampling criteria.

After each invitation or reminder email sent out, error messages were collected and dealt with where possible. This included documenting the number of server rejections and automated replies, as well as the response status of the study subjects. The survey was ‘closed’ (ie, no further responses accepted) on 16 March 2007. Following the close of the survey, we audited the response data, analyzing the factors that affected the numerator and denominator in the ultimate determination of response. We also conducted a sensitivity analysis to account for the inherent uncertainty associated with the impact of some of the factors on the response rates. Ethics approval for the survey was provided by the University of Toronto’s Research Ethics Board.

## Results

The determination of the response rate requires both a numerator and a denominator. The numerous factors affecting the determination of both are described below.

### The Numerator

The response rate’s numerator varies and can represent the number of study subjects who viewed, participated, or completed the survey. There were two main factors which influenced the determination of the numerator: duplicate/unattached responses and response completeness.

#### Duplicate/Unattached Responses

 With the list management feature and the distribution/collection options used, the potential for duplicate responses was high. A duplicate response could occur if the intended study subject accessed the survey through the individual-specific Web link more than once (eg, each click on the Web link resulted in a separate response). A duplicate response could also occur if the intended study subject forwarded the invitation or reminder emails to another individual who then accessed the survey through the same individual-specific Web link. In either case, a new response associated with the original study subject’s email address would be automatically captured and added to the data set. While a duplicate response may often reflect benign intentions (eg, not having time to complete the survey in one sitting or sending the email invitation from a work email address to a personal email address), researchers need to be aware of attempts to influence the results by essentially ‘stuffing the ballot box’.

Of the 2031 responses captured in the database, 1699 were associated with a single email address representing an intended study subject from the original sample. There were 321 responses captured of which two or more were associated with the same study subject’s email address. Another 11 responses captured were not associated with any study subject’s email address which, based on the list management and response/collection options used, should not have been possible.

For the numerator, criteria for what to do with duplicate and unattached responses need to be established. The unattached responses, while raising some lingering questions regarding the accuracy of the list management feature, represented a very small proportion of the sample. Therefore, the 11 responses not associated with a study subject were excluded. Criteria for exclusion of duplicate responses represent a more challenging problem that includes inherent uncertainty and requires judgement. For the 321 duplicate responses, 171 were ultimately excluded based on the exclusion criteria set out in Table 1. Overall, there were 1849 responses associated with a unique email address.

**Table 1:** Duplicate/unattached response exclusion criteria

Exclusion criteria	Responses excluded (N)
(i) When a response is not associated with a study subject (ie, no email address), the response is excluded.	11
(ii) When only 1 of 2 or more responses associated with the same study subject (ie, a unique email address) indicate in the first question of the survey that the invitation email was received directly from csi.survey@cancercare.on.ca, the other responses are excluded.	33
(iii) When 2 or more responses associated with the same study subject (ie, a unique email address) are ‘identical’, the initial response is included and all subsequent responses are excluded.	12
(iv) When 2 or more responses associated with the same study subject (ie, a unique email address) are ‘identical’ up until a certain question, after which 1 response continues on and the other responses are incomplete, the less complete responses are excluded.	112
(v) When 2 or more responses associated with the same study subject (ie, a unique email address) are clearly different (eg, responses indicate different sex, position, location of work, etc.), the most unlikely responses (eg, based on comparison to available demographic/position information for the study sample) are excluded.	4
(vi) When none of the above criteria apply, multiple responses associated with the same study subject (ie, a unique email address) were randomly selected to exclude all but 1 response per study subject.	10
**Total responses excluded**	**182**

#### Response Completeness

Response completeness reflects varying response patterns and reporting options that can influence the determination of the numerator [10]. Simply clicking on the survey Web link in the invitation email is defined as a response by SurveyMonkey.com. For this survey, the crude number of responses captured was 2031, with 1849 unique respondents ‘viewing’ the survey after adjusting for duplicate responses. The number of views, however, does not necessarily reflect participation, as no questions need to be answered.

A more conservative measure of response completeness links ‘participation’ to an actual response to a specific question. In this survey, the tenth question on the survey asked the respondent to identify the regional cancer program most relevant to their clinical/professional work. As this question was the base for the conditional logic that directed the respondent to region-specific survey sections, a response to this question was required before the respondent could move on to subsequent sections of the survey. For this survey, adjusted for duplicate responses, 1769 unique respondents participated in the survey by answering the tenth question.

As not all ‘participants’ completed the remaining 54 Likert scale items in the survey, another measure of response completeness reflects the number of respondents who ‘complete’ the final question on the survey. In this survey, adjusted for duplicate responses, 1616 respondents completed the final survey question (although it should be noted that only 722 respondents completed all survey questions).

**Figure 1:**
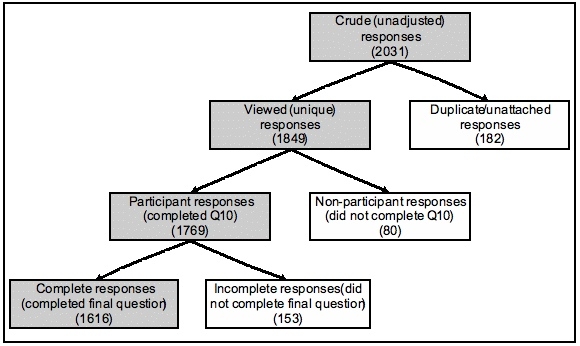
Estimating the numerator

Therefore, the response data collected for this survey provide four plausible numerators that could be reported (Figure 1). If we exclude the crude number of responses that is not adjusted for duplicate/unattached responses, there are still considerable differences between the number of respondents who viewed (n = 1849), participated (n = 1769) or completed (n = 1616) the survey.

### The Denominator

The response rate’s denominator varies and can represent the number of individuals who received, or were intended to receive, an invitation to participate in the survey. There are a number of factors impacting on the denominator that require consideration including forwarding of the invitation email to unknown individuals, server rejections, automated replies, spam filters, and ‘opt in/out’ options. These factors are elaborated in turn below.

#### Forwarding Email Invitation to Unknown Individuals

Forwarding of invitation emails affects the number of individuals who actually receive the invitation. While the list management and response/collection options provide greater certainty with respect to the impact of forwarding on the numerator, it is much more difficult to assess the impact on the denominator.

For this survey, there were many indications that forwarding was occurring. For example, when responding to the first question of the survey, 47 individuals indicated they did not receive the invitation email directly from csi.survey@cancercare.on.ca, but rather received the message from another individual (eg, colleague/friend). The many duplicate responses associated with the same study subject also suggested that invitation/reminder emails were being forwarded to other email addresses. While email forwarding leads to an increase in the denominator, there is no clear mechanism to determine how many invitation emails were actually forwarded to unintended recipients. Unfortunately, there is little guidance on how the denominator should be adjusted, if at all, to acknowledge the impact of forwarding. There are two main options which include (a) not adjusting the denominator but rather adjusting the numerator (ie, remove duplicate responses that are due to email forwarding) to maximize the likeliness that a response was from an intended study subject or (b) adjust the denominator based on an estimate of the number of email invitations that were forwarded to other individuals. Therefore, as part of our sensitivity analysis, we considered the impact on the denominator if 1% (56/5636), 5% (282/5636), or 10% (564/5636) of original email invitations were forwarded to other individuals.

#### Server Rejection (Bounce-Back)

Server rejections usually represent an email that does not reach the intended recipient and normally result in a bounce-back email to the original sender which provides information on the reason for the rejection. Server rejections were monitored after the initial email invitation and after each of the 3 subsequent reminder emails. Some server rejections were due to incorrect or dormant email addresses, while other rejections were due to temporary (eg, communications failure, message delays, disabled mailbox, etc.) and permanent (eg, no such recipient, syntax/format error, etc.) delivery failures. For the invitation and 3 reminder emails, 346 study subjects (ie, a unique email address) had at least 1 server rejection.

Incorrect email addresses were updated and invitation emails resent where possible. We confirmed that 171 of these study subjects were no longer in their positions. However, there was little guidance for how to deal with other temporary or permanent server rejections. Examining each server rejection individually, we noted that for 54 study subjects, all attempts to send the invitation and reminder emails were rejected by the server. For another 19 study subjects, 3 of the 4 attempts were rejected by the server, while for 25 study subjects, 2 of the 4 attempts were rejected, and for 77 study subjects, 1 of the 4 attempts was rejected (Table 2).

In the case where all invitation/reminder emails to a study subject are rejected by the server, should that study subject be removed from the sample, thereby reducing the denominator? Complicating this issue, our response audit showed that for 2 of the 54 study subjects who had all 4 invitation/reminder emails rejected by the server, a response had been captured. Ultimately, we excluded those study subjects that did not receive a single invitation or reminder email (ie, all email invitation/reminders were rejected by the server), adjusted for those where a response was still captured.

**Table 2:** Server rejection and automated reply patterns

Server rejection patterns (unadjusted)	Responses (N)	Automated reply patterns (unadjusted)	Responses (N)
Total invitation emails sent out^*^	5640	Total invitation emails sent out^*^	5640
No server rejections (0/4)	5294	No automated replies (0/4)	5344
Server rejection to 1/4 launch/reminder emails	77	Automated reply to 1/4 launch/reminder emails	198
Server rejection to 2/4 launch/reminder emails	25	Automated reply to 2/4 launch/reminder emails	63
Server rejection to 3/4 launch/reminder emails	19	Automated reply to 3/4 launch/reminder emails	12
Server rejection to 4/4 launch/reminder emails	54	Automated reply to 4/4 launch/reminder emails	2
Server rejection – confirmed no longer at organization	171	Automated reply – extended leave	21

^*^Includes 4 additional invitations sent out after initial survey launch

#### Automated Reply (Out-of-Office)

Automated reply options available in most email software programs (eg, Microsoft Outlook) allow an individual to set up a message that is automatically sent in reply to all received email messages over a specified period of time. A key distinction between server rejections and automated replies is that server rejections normally indicate that the email did not reach its intended target, while automated replies normally indicate that the email was received, but that the intended recipient may not have had the opportunity to read and/or respond to the message.

The most common example of an automated reply is the out-of-office reply. Whether an out-of-office reply is received by the original sender is affected by a range of settings, with some providing an automated reply only to the first email received from a unique address within a specified period and others restricting to whom automated replies will be sent (eg, only to emails originating from within the individual’s organization or from specified ‘safe’ domains). Therefore, it is likely that not all out-of-office replies are received by the survey sender. Fortunately, for those out-of-office replies that are received, they usually indicate the duration of an individual’s absence.

For this survey, one or more automated replies to the invitation/reminder emails were received from 296 study subjects (Table 2). Of these, 23 indicated that the study subject would be out-of-office for the duration of the survey (ie, either an automated reply indicating an extended leave for the period of the survey or automated replies to each of the 4 invitation/reminder emails). This type of information is not usually available to researchers when using traditional survey modalities. Therefore, in terms of determining response, there is limited guidance on how to use it. Should those individuals who clearly indicate that they will be away for the duration of the survey be excluded from the denominator? Again complicating the issue, our response audit revealed that a response was captured for 3 of the 23 study subjects whose automated replies indicated they would be out-of-office for the duration of the survey. Ultimately, we excluded those study subjects where an out-of-office reply was received for each email invitation/reminder or indicated in the automated reply that they would be away for the duration of the survey, adjusted for those where a response was still captured.

#### Spam Filters (Junk Mail)

Spam filters present another degree of uncertainty in terms of determining the denominator. There are two main approaches to dealing with spam. The first involves preventing potentially unwanted emails from reaching the email server, and the second involves automated marking of potentially unwanted emails as spam and allowing individuals to review and filter them accordingly.

The former approach, often used by large organizations such as hospitals and universities, involves commercially available services. However, while these filters do a reasonably good job of detecting spam, they also potentially filter out emails that are not spam, which therefore never reach the intended individual. Some of the commercially available spam filter services guarantee false positive rates of 1 in 10,000 or better (eg, MessageLabs claims a false positive rate of 1 in 333,333 [19]), which for our survey would suggest that it would be unlikely that the invitation or reminder emails would be filtered as spam. Furthermore, SurveyMonkey.com’s list management feature allowed us to designate the email address from which the invitation and reminder emails would be sent. Therefore, we used the provincial cancer agency’s domain (ie, ‘cancercare.on.ca’) for the invitation email, which, as a recognizable domain within the Ontario health care system, should have reduced the possibility that the invitation/reminder emails would have been filtered as spam. Although, we did not have information on the specific commercial spam filter services used by the study subjects’ organizations, when at least one response was received from an organization’s email domain, we could deduce that the specific organization’s spam filter did not automatically filter out the email invitations sent to study subjects with the same organization’s email domain.

The impact of the latter spam filtering approach is less clear. Most email providers and software programs provide some type of spam filter control for the individual user. This includes a number of filtering levels that range from blocking all emails from an email address not designated in a safe list to allowing almost all but the most obvious spam to the inbox. There are also customizable filtering options, where user-defined keywords can be filtered out automatically. For example, if an individual sets their filter to exclude any emails with the word ‘survey’ in the message, our invitation email would not be received.

In contrast to email forwarding, where the concern is that more individuals receive the invitation email than intended, the concern with spam filters is that not all study subjects receive the invitation email. Unfortunately, it is very difficult to determine the extent to which spam filters affect the number of study subjects who actually receive the invitation. Ultimately, for our best estimate, we did not adjust the denominator to account for the impact of spam filters. However, similar to our assessment of the impact of email forwarding, we did consider the impact on the denominator if 1% (56/5636), 5% (282/5636), or 10% (564/5636) of invitation emails were filtered from the intended recipients, as part of the sensitivity analysis.

#### ‘Opting Out’ and ‘Opting In’

When using the list management feature, Surveymonkey.com requires that an ‘opt out’ Web link be included in the invitation email message. In part to prevent use of the list management feature for distributing spam, the opt out option allows those individuals sent the invitation email to click on the specified opt out link which removes that individual’s email address from the list and prevents further emails from being sent to that individual. It should be noted that when an individual decides to opt out, it may not be possible to send an email to their email address for an extended period of time (eg, at least 1 year in our most recent experience with SurveyMonkey.com’s list management feature).

For this survey, we had 126 individuals actively opt out using the provided Web link. Another 18 individuals contacted the study’s research coordinator to indicate that they should not have been included in the sample. A reasonable explanation for why they should not be included in the survey was provided by 11 of these individuals (eg, no longer a cancer care provider). From the denominator, 9 were removed while 2, having already initiated their response, were included. The remaining 7 individuals were considered eligible recipients and were therefore also included in the denominator.

There were also 4 individual requests to be added to our sample. The case for each individual was reviewed and accepted, with an invitation email then sent to each to allow direct access to the survey. This reflects an ‘opt in’ option, which thereby increased the denominator.

### Estimating the Denominator

**Figure 2:**
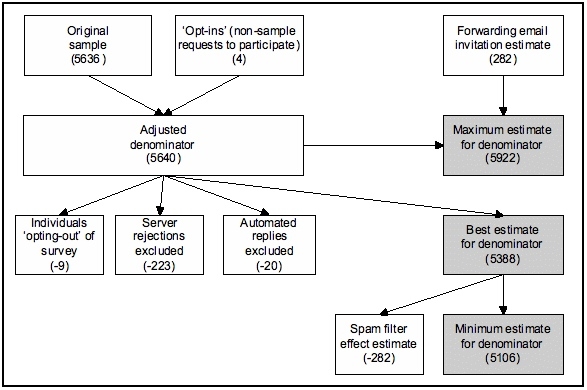
Estimating the denominator

Considering the factors described above, there is considerable uncertainty inherent in any estimate of the denominator for an Internet survey. Most factors, such as server rejections, automated replies, and spam filters, tend to reduce the number of individuals receiving the invitation email, while other factors, such as email forwarding, tend to increase the number of individuals receiving the invitation email. Figure 2 presents the impact of various factors for estimating the denominator.

### Response Rate Estimates and Sensitivity Analysis

While established protocols for determining response rates for mail/telephone surveys exist [8], these survey modalities lack the type of tracking data immediately available for Internet surveys. Therefore, one needs to ask whether response rates should be calculated differently for Internet surveys. Based on our response audit, Table 3 sets out a maximum, minimum, and best estimate for both the numerator and denominator. From these estimates, a sensitivity analysis was conducted that examines the impact of the various assumptions on the overall response rate. Based on our best estimates, the response rate was 32.8%. However, under more or less conservative assumptions for the numerator and denominator, the sensitivity analysis suggests that the response rate could have varied by more than 12% (27.3% to 39.8%). This is based on a 5% estimate for the email forwarding and spam filter effect. When considering the impact of lower (1%) or higher (10%) estimates for the email forwarding and spam filter effects, the sensitivity analysis suggests that the response rate could have varied by more than 9% (28.4% to 38.1%) with a lower (1%) estimate of effect, or by more than 16% (26.0% to 42.1%) with a higher (10%) estimate of effect.

**Table 3:** Response rate estimates and sensitivity analysis

			Maximum estimate				Best estimate			Minimum estimate		
**Numerator**												
Crude			2031				2031			2031		
Duplicate/unattached			0				-182			-182		
Response completeness (view – participate)			0				-80			-80		
Response completeness (participate – complete)			0				0			-153		
** Numerator Estimate**			**2031**				**1769**			**1616**		

**Denominator**												
Crude			5636				5636			5636		
Opt in			+4				+4			+4		
Forwarding impact^*^			+282				0			0		
Opt out			0				-9			-9		
Server rejection			0				-223			-223		
Automated reply			0				-20			-20		
Spam filter effect^*^			0				0			-282		
** Denominator Estimate**			**5922**				**5388**			**5106**		

**Numerator**	**Crude**	**Max**		**Max**	**Max**	**Best**		**Best**	**Best**	**Min**	**Min**	**Min**
	2031	2031		2031	2031	1769		1769	1769	1616	1616	1616

**Denominator**	**Crude**	**Max**		**Best**	**Min**	**Max**		**Best**	**Min**	**Max**	**Best**	**Min**
	5640	5922		5388	5106	5922		5388	5106	5922	5388	5106

**Response rate**	36.0%	34.3%		37.7%	39.8%	29.9%		32.8%	34.6%	27.3%	30.0%	31.6%

^*^based on 5% (282/5636) estimate of effect; max = maximum estimate; best = best estimate; min = minimum estimate

## Discussion

Surveying a specified sample of health care providers and administrators, we intended to use an Internet survey to replicate, to the extent possible, traditional survey modalities (eg, mail/telephone). However, given the range of design, distribution, and response/collection options available, Internet surveys present unique features that affect the determination of response rates. This response audit raises important questions for researchers regarding the appropriateness of traditional rules/protocols used for determining and reporting on the response of health care providers and administrators to Internet surveys.

We concur with the CHERRIES statement recommending more complete and detailed descriptions of the conduct of Internet surveys [10]. While much of the CHERRIES checklist is relevant to all Internet surveys, there is less emphasis on the use of commercially available canned software programs, such as SurveyMonkey.com, targeted to specified (vs convenience) samples. Our response audit suggests there is an equally strong need for detailed information on how Internet surveys directed to specified samples using commercially available software programs are administered, how response rates are calculated, and how multiple responses from the same individual are prevented. The distribution/list management and response/collection options available from these Internet survey vendors should be clearly identified and the particular options selected should be justified. It would be helpful if Internet survey vendors provided more detailed information on the effects of the various options on respondent access, privacy, and confidentiality as well as data capture, but without this, researchers should provide details of any pre-testing of the distribution/list management and response/collection options available. Reports of Internet surveys should also include discussion of assumptions used in determining response rates, including the impact of email forwarding, server rejections, automated replies, and spam filters.

Some limitations to this work should be noted. First, this response audit was based on a survey conducted through a widely available Internet survey vendor (SurveyMonkey.com) in February 2007. However, SurveyMonkey.com has since modified its response/collection options, highlighting the evolving nature of this field. Further, while Internet survey options may be similar across the many competing vendors, standards for distribution/list management and response/collection options, as well as the type of response status data collected, differ. While the underlying factors that impact on response rates for Internet surveys are the same, there is a need to further standardize and categorize the necessary descriptive information that should be reported for any Internet survey. Second, it should be noted that a defining characteristic of a subset of our target population was the need to respond from a shared computer. This influenced our choice of the response/collection option selected, which allowed more duplicate responses than would otherwise have been the case. Third, our sensitivity analysis of the effects of email forwarding and spam filters were based on rough estimates. While our intent was to use fairly conservative estimates to highlight the potential impact of these factors, there is a need for more work in this area to accurately measure the effect of email forwarding and spam filters on response rates. Fourth, although not unusual for surveys of clinicians [20], the overall response rate to our survey (however determined) was low. However, for the purposes of the response audit, our large sample of over 5000 study subjects provided sufficient data from which to examine the factors that influence response rates. Lastly, this response audit focuses on technical factors relevant to Internet surveys that impact on response rates. We acknowledge that other factors such as respondent characteristics may also influence response; however, this does not preclude the need to accurately describe how Internet surveys are conducted and response rates calculated.

While it has been questioned whether Internet surveys will ever become part of mainstream research [17], it seems clear that Internet surveys are here to stay. Therefore, there is a growing need to improve the documentation and reporting of Internet survey design features, distribution, and response/collection options employed. As Internet survey options continue to evolve, further consideration of the way survey research is conducted and reported is needed. The CHERRIES statement is an important starting point [10]; however, further emphasis on the use of commercially available Internet survey products for specified, rather than convenience samples, is needed. We hope this paper advances development in this important methodological area.
